# Impulsive Social Influence Increases Impulsive Choices on a Temporal Discounting Task in Young Adults

**DOI:** 10.1371/journal.pone.0101570

**Published:** 2014-07-02

**Authors:** Jodi M. Gilman, Max T. Curran, Vanessa Calderon, Luke E. Stoeckel, A. Eden Evins

**Affiliations:** Center for Addiction Medicine, Massachusetts General Hospital Department of Psychiatry, Harvard Medical School, Boston, Massachusetts, United States of America; University of Chicago, United States of America

## Abstract

Adolescents and young adults who affiliate with friends who engage in impulsive behavior are more likely to engage in impulsive behaviors themselves, and those who associate with prosocial (i.e. more prudent, future oriented) peers are more likely to engage in prosocial behavior. However, it is difficult to disentangle the contribution of peer influence vs. peer selection (i.e., whether individuals choose friends with similar traits) when interpreting social behaviors. In this study, we combined a novel social manipulation with a well-validated delay discounting task assessing impulsive behavior to create a social influence delay discounting task, in which participants were exposed to both impulsive (smaller, sooner or SS payment) and non-impulsive (larger, later or LL payment) choices from their peers. Young adults in this sample, n = 51, aged 18–25 had a higher rate of SS choices after exposure to impulsive peer influence than after exposure to non-impulsive peer influence. Interestingly, in highly susceptible individuals, the rate of non-impulsive choices did not increase after exposure to non-impulsive influence. There was a positive correlation between self-reported suggestibility and degree of peer influence on SS choices. These results suggest that, in young adults, SS choices appear to be influenced by the choices of same-aged peers, especially for individuals who are highly susceptible to influence.

## Introduction

Epidemiological studies have demonstrated that adolescents and young adults are more likely to exhibit a range of behaviors if they affiliate with friends who also engage in those behaviors. Adolescent alcohol use (see [1] for review), cigarette smoking, illegal drug use (e.g. [2]), and aggressive behavior (e.g. [3]), are all associated with peer engagement in these activities. Prosocial peer behavior is predictive of such behavior as alcohol abstinence [4], and reduced violent behavior [5]. The exact nature of these associations, however, is unknown. Because people often choose friends who engage in similar types of behavior as themselves [6], it is unclear whether these young adults engage in behaviors that they otherwise would not engage in if it weren't for their social environment.

Socialization effects have been shown to influence people to engage in the behaviors that they observe around them [7], [8], but controlled laboratory studies of these effects are rare in the literature. Objective laboratory experiments are necessary to quantitatively assess susceptibility to social influence on impulsive behavior, and could be useful in investigating whether certain subtypes of people are especially likely to have their behavior influenced by their peers. Further, a well-controlled laboratory task assessing behavioral susceptibility to peer influence could be critical to assessing this effect, as research participants have been shown to both under- and over-report these behaviors in self-report scales (see [9] for review).

Delay discounting is a well-validated, laboratory-based paradigm, conceptualized as an operational measure of impulsive behavior [10], that can allow us to test whether social influence affects behavior. In a typical delay discounting paradigm, participants are presented with a series of choices between small, sooner (SS) monetary rewards, and larger, later (LL) rewards. The value of a reinforcer is diminished or ‘discounted’ by the delay at which it is received [11]. The degree to which rewards are diminished by delay, termed discount rates, are reliable predictors of decision-making behavior, with greater discount rates characterizing some clinical populations. People with drug and alcohol dependence (see [12] for review), obesity [13], [14], and problem gambling [15] devalue or discount delayed rewards to a greater extent than do those in the general population. Although discount rates have high test-retest reliability [16], stability across time [17] and commodities within subjects, [18], and have a biological basis [19], discount rates do appear to be modifiable, to some extent by reward magnitude, transcranial magnetic stimulation, framing effects, and acute drug effects (see [20] for review). Thus, abnormal delay discounting may present a target for therapeutic intervention.

In delay discounting studies, participants are usually instructed to make choices for SS or LL rewards without social influence on their decision. In reality, we live in a social world, where many of our decisions are affected by the reactions or valuations of those around us [21]. The choices that other people make affect the choices that we ourselves make, as we are susceptible to persuasive arguments [22], [23], [24], [25], to desire for conformity with peers [26], [27], [28], [29], and to acquired and sometimes subconscious norms and cultural values [30], [31]. Social influence is just beginning to be studied as a factor in impulsive decision-making, and research has recently emerged demonstrating that in a delay discounting task, adolescents display a greater preference for SS rewards in the presence of both known [32] and unknown [33] peers. Peer influence on impulsive decision-making is particularly important to study in adolescent/young adult populations, as this developmental stage is associated with high susceptibility to peer influence [34], steeper discounting rates, and greater impulsive decision-making compared to older adults [35].

We hypothesized that the choices of young adult participants would be influenced by the specific choices of peers, such that choice of SS rewards over LL rewards could be both increased and decreased depending on whether participants are informed that peers have chosen SS or LL rewards. To test this hypothesis, we designed a delay discounting task with a novel social influence manipulation, in which young adult participants performed a standard delay discounting task while being exposed to impulsive and non-impulsive influence. If social influence can indeed affect preferences for rewards, this would indicate that impulsive decision making can be directly modulated by social influence in young adults.

## Method

### Participants

Fifty-one young adults (28 men, 23 women), ages 18–25 (mean age  = 20.7, SD = 1.94) participated in this study. Participants were medically healthy, with no history of psychiatric disorders (verified by the Structured Clinical Interview for DSM-IV (SCID) [36]. Participants completed a written, documented informed consent form prior to initiation of study procedures. The consent procedure and all study procedures were approved by the Partners Human Research Committees (PHRC) (consisting of the Institutional Review Boards (IRBs) of the Brigham and Women's/Faulkner Hospital, Massachusetts General Hospital (MGH), McLean Hospital and North Shore Medical Center (NSMC)).

### Study Procedures

Prior to participation, participants were told that they were participating in a study on judgment and decision-making, but were not told that the study assessed social influence. Before beginning the delay discounting task, participants were handed a binder of 32 color photographs (16 of each gender) taken from the Texas Center for Vital Longevity at University of Texas, Dallas (happy expressions; ages 18–29) [37] and the Max Plank FACES database (happy expressions, young adults age 19–31) [38]. The following script was read:

*We are going to show you photos of other people who have participated in this experiment. In some of the games you'll play, you will get to see their answers. Please choose people whose answers you would like to see.*

**Figure 1:**
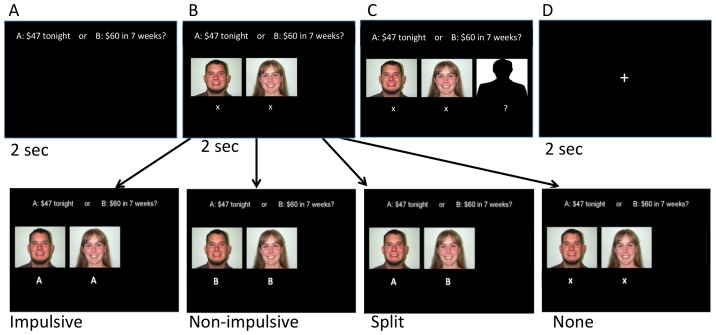
Depiction of Social Influence Manipulation. After showing participants their option (**Panel A**), four types of influence could be presented (Impulsive, Non-impulsive, Split, and None) (**Panel B**), as shown in the lower panel. Participants were then asked to make their choice. (**Panel C**) and then had a 2 second rest between trials (**Panel D**). Please see Methods for detail.

The photographs chosen by participants (hereafter referred to as ‘peers’) were then presented during the task (see Fig 1). To increase believably of the paradigm, we asked the participants if we could take their photographs to be added to the binder of previous participants. (Please note, photographs of participants were not actually shown to subsequent participants; all photographs presented were from the databases listed above). Participants completed a variety of other self-report and behavior tasks as part of a larger study on social influence and decision-making in a number of domains (other results of this larger study have not yet been published).

After completing study procedures, participants were debriefed, and were given the option of removing their photograph from the database.

### Questionnaires

Participants completed the Multidimensional Iowa Suggestibility Scale (MISS) [39], which assesses susceptibility to influence in five domains: consumer suggestibility (suggestibility to commercials, products), persuadability (changing one's mind based on other peoples' arguments), physiological suggestibility (feeling cold when someone else is shivering), physiological reactivity (feeling jumpy after watching a scary movie), and peer conformity (liking the same celebrities/fashion/music as friends) whose subscales were summed to compute a total suggestibility score.

Participants also completed The Barratt Impulsiveness Scale (BIS-11; [40]), a questionnaire designed to assess the personality/behavioral construct of impulsiveness.

### Delay Discounting Task

The delay discounting task used was a computerized version of the Kirby Monetary Choice Questionnaire [41]. Participants were presented with a choice of (A), the SS reward or (B), the LL reward, of varying amounts. The experiment is illustrated in Fig 1. First, participants were shown a screen presenting two options, a SS and a LL reward, for 2 seconds. Next, participants were shown the ‘choices’ of two of the previously selected peers with their photographs for two seconds. There were four types of influence: Impulsive (both peers chose A, the SS reward), Non-impulsive (both peers chose B, the LL reward), Split (one peer chose SS, one chose LL), and, as a control condition, None (responses of peers were not revealed to the participant). Next, participants were instructed to make their choice. A crosshair was then presented for 2 seconds. This was repeated 84 times. As in the Kirby questionnaire, there were 21 choices between SS vs LL rewards. The 21 choice trials define 20 bounded ranges of discounting parameter values [41]. Each of the 21 combinations was repeated four times, once with each type of influence (Impulsive, Nonimpulsive, Split, and None), in a randomized, counterbalanced order. In total, participants made 84 choices (21 multiplied by the 4 types of influence). The experiment took approximately 10 minutes to complete. Participants were told that these choices were hypothetical; no reward was actually given, as hypothetical and actual rewards produce similar patterns of discounting [42], [43].

### Dependent Variables

For each of the influence conditions of interest (no influence, impulsive influence, and non-impulsive influence), two parameters were calculated. First, we calculated the percentage of SS choices that the participant selected out of the 21 choice trials in each influence condition. This parameter captured the total number of SS choices with each category of influence, regardless of the consistency of impulsive responding. This measure captured all SS choices, regardless of general patterns of responding, and was therefore sensitive to random or occasional SS choices that may have been outside of the participants' general patterns.

Second, we calculated each individual's discount rate, or *k* value, based on methodology described in [41]. Discount rates were calculated assuming a hyperbolic discounting model; a higher *k* value indicates steeper discounting of a delayed reward.

Due to the pseudo-randomized nature of the questions across the influence types, *k* values for each subject could not be assigned by simply finding a single switch point from SS to LL choices. Instead, *k* values were assigned to subjects by computing the proportion of responses consistent with each *k* value and assigning the subject the value yielding the highest consistency [41]. A consistent response was defined as an SS choice with a *k* value less than the subject's assigned *k* value, or conversely an LL choice with a k value greater than the subject's assigned *k* value. Using this method, we were also able to acquire a measure of how consistent the assigned value was across all of the individual participant's responses. This consistency measure was defined as the number of consistent choices divided by the total number of possible choices (21 per influence type). For example, a subject who was assigned a *k* value of 0.0042 but who also chose the LL reward on a question with a lower *k* value would have one inconsistent response. We accepted consistency scores of 0.75 or higher for all analyses of *k* values.

Because it is not known whether impulsive social influence could (a) influence participants to make more random or occasional SS responses throughout the experiment that may have been inconsistent with their usual pattern of decision-making, or (b) have a consistent effect on decision-making, shifting choices consistently toward the SS rewards, we investigated the effect of influence on both percent of impulsive choices and *k* values. *K* values were log_10_ transformed [41], [44] due to the right skew of the k distribution. Larger log[*k*] values (closest to −1) indicate a steeper discount function indicative of consistently more impulsive choices.

### Statistical Analyses

Percent of SS choices and log[*k*] values were entered as dependent variables into separate repeated-measures ANOVAs, with influence type as the independent variable. If a significant *F* value was detected in the ANOVA, pairwise comparisons were examined using Tukey's test for multiple comparisons. Effect sizes and 95% confidence intervals were computed using Prism 6 software (GraphPad Software, Inc).

To determine whether the effect of social influence on percentage of SS choices or *k* values was related to self-reported susceptibility to influence, we calculated a difference score for each participant on each parameter (i.e. percentage SS choices and log[*k*] values during *impulsive* influence minus percentage SS choices and log[*k*] values during *non-impulsive* influence). These difference scores were then correlated with participants' total scores on the MISS questionnaire (total susceptibility) and the subscale “peer conformity,” as this subscale was most relevant to our hypotheses.

To determine whether performance on the modified delay discounting task was correlated with the personality/behavioral construct of impulsiveness, we ran correlations between BIS total scores and both percentage of SS choices and log[*k*] values in the “no influence” conditions.

Finally, in order to investigate the role of individual differences in self-reported susceptibility and task behavior, we performed a median split based on suggestibility scores on the MISS. We performed an ANOVA examining the percent of impulsive choices in each task condition (impulsive, non-impulsive, and no influence) in “high-suggestibility” participants (those scoring in the top half on the MISS; n = 25), and in the “low suggestibility” participants (those scoring in the bottom half on the MISS; n = 25).

## Results

Out of 51 participants, *k* values could not be calculated for 4 participants because they chose the delayed choice every time (Table S1). Additionally, 3 participants had consistency scores below 0.75, indicating that the parameter estimated for the *k* values would not be a good fit. Therefore, analyses of *k* values were calculated in 43 participants.

### Peer Influence on Percentage SS Choices

**Figure 2:**
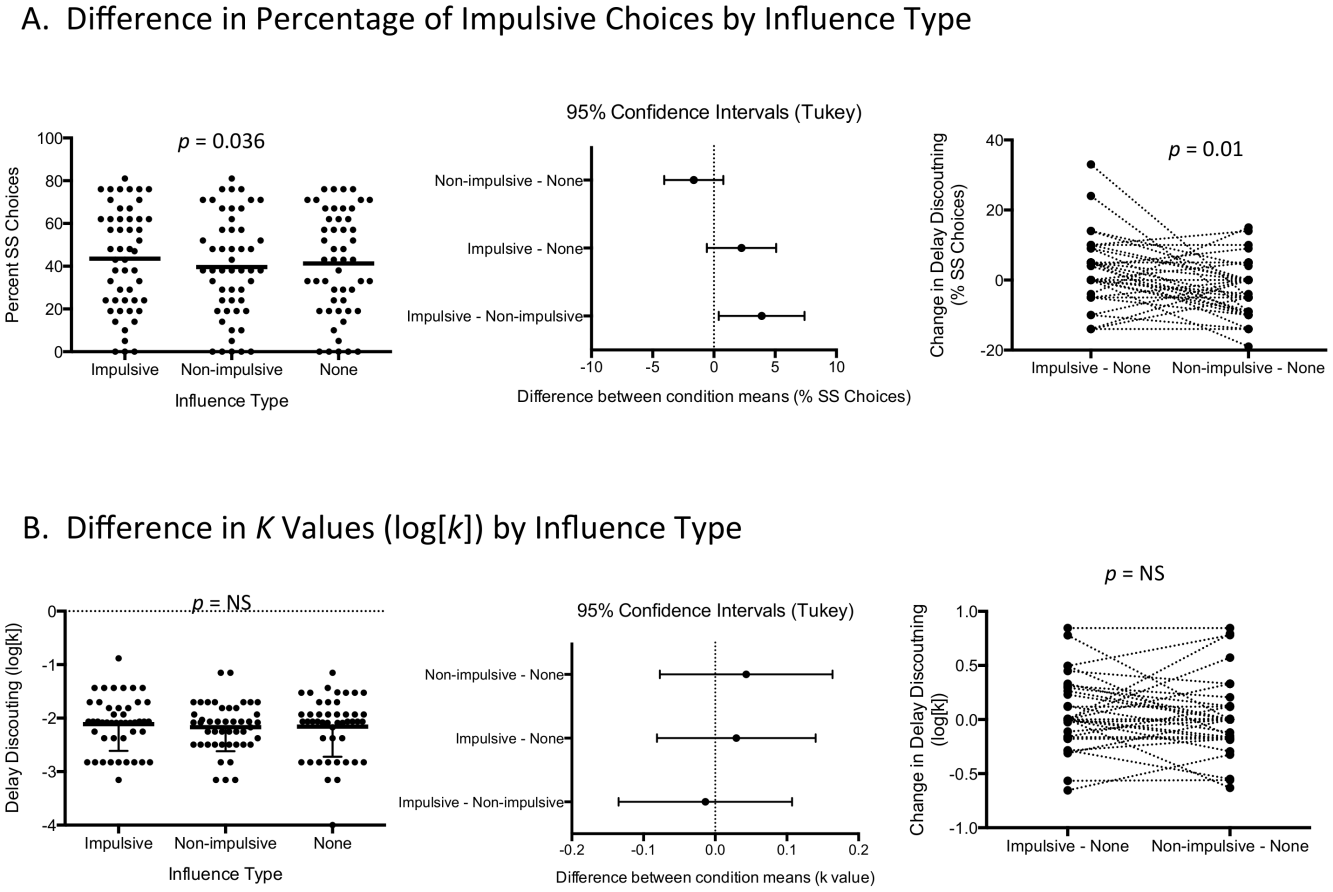
Percentage impulsive choices and k values by influence type. **Panel A and B, Left.** Each point represents an individual participant's percent of SS choices (top panel), or k value (bottom panel) after exposure to each of influence type. The horizontal bar shows the mean of the group during each influence type. **Middle**. Each bar shows the 95% confidence intervals of the difference between the means of each pair of conditions in percent of SS choices (top panel), or k value (bottom panel). The points represent the mean of each difference. Impulsive influences are generally shifted to the right, indicating that impulsive responding increased under impulsive influence conditions. **Right**. Each point and dotted line represents an individual participant's percent of SS choices (top panel), or k value (bottom panel) after exposure to impulsive influence (minus no influence ‘none’) and non-impulsive influence (minus ‘none’). Paired t-tests indicate a significant difference of influence type on percentage of SS choices, and a non-significant effect on k values.

A one-way ANOVA indicated that peer influence had a significant effect on percent of SS choices (*F* (2,50) = 3.55, *p* = 0.036, ηp^2^ = 0.127). Post-hoc tests indicated that participants had a higher rate of SS choice in decisions with impulsive influence than with non-impulsive influence (*p* = 0.03; Cohen's *dz* = 0.38; Table 1; Figure 2A). Small effect sizes were detected when comparing the rate of SS choices between the impulsive influence vs. the no influence condition, and the nonimpulsive vs. no influence condition, but these comparisons were not significant (Table 1).

**Table 1:** Comparisons in Influence Conditions in All Participants.

Pairwise Comparisons Influence Type	Effect Size (Cohen's *dz*)	Mean Diff.	95% CI of diff.	Adjusted *p* Value
***Percentage of Impulsive Choices***				
Impulsive vs. Nonimpulsive	**0.38**	3.9	[0.40–7.40]	**0.03**
Impulsive vs. None	**0.27**	2.3	[−0.57–5.08]	0.14
Nonimpulsive vs. None	**0.23**	−1.6	[−4.06–0.76]	0.23
***Log[k]***				
Impulsive vs. Nonimpulsive	0.04	−0.01	[−0.13–0.11]	0.96
Impulsive vs. None	0.09	0.03	[−0.08–0.14]	0.79
Nonimpulsive vs. None	0.13	0.04	[−0.07–0.16]	0.66

All *p* values were corrected using Tukey's multiple comparison test. Cohen's *dz* values >0.2 and *p* values <0.05 are indicated in bold text.

To control for the ‘no influence’ condition, we subtracted percent SS choices with ‘impulsive’ and ‘nonimpulsive’ influence from the no influence control condition (i.e. (impulsive – none) and (nonimpulsive – none). Paired *t*-tests indicated a significant difference between impulsive and nonimpulsive influence, when subtracted from choices made with no influence, on impulsive decision-making (*t*(50) = 2.69, *p* = 0.01; Cohen's *dz* = 0.38; Figure 2A).

### Peer Influence on Log[k] Values

Log[*k*] values were not significantly different across conditions. There was no difference between (impulsive – none) and (nonimpulsive – none) conditions, controlling for the ‘no influence’ condition, in paired *t*-tests (Figure 2B).

### Association of Delay Discounting Performance with Self-Reported Susceptibility to Peer Influence

**Figure 3:**
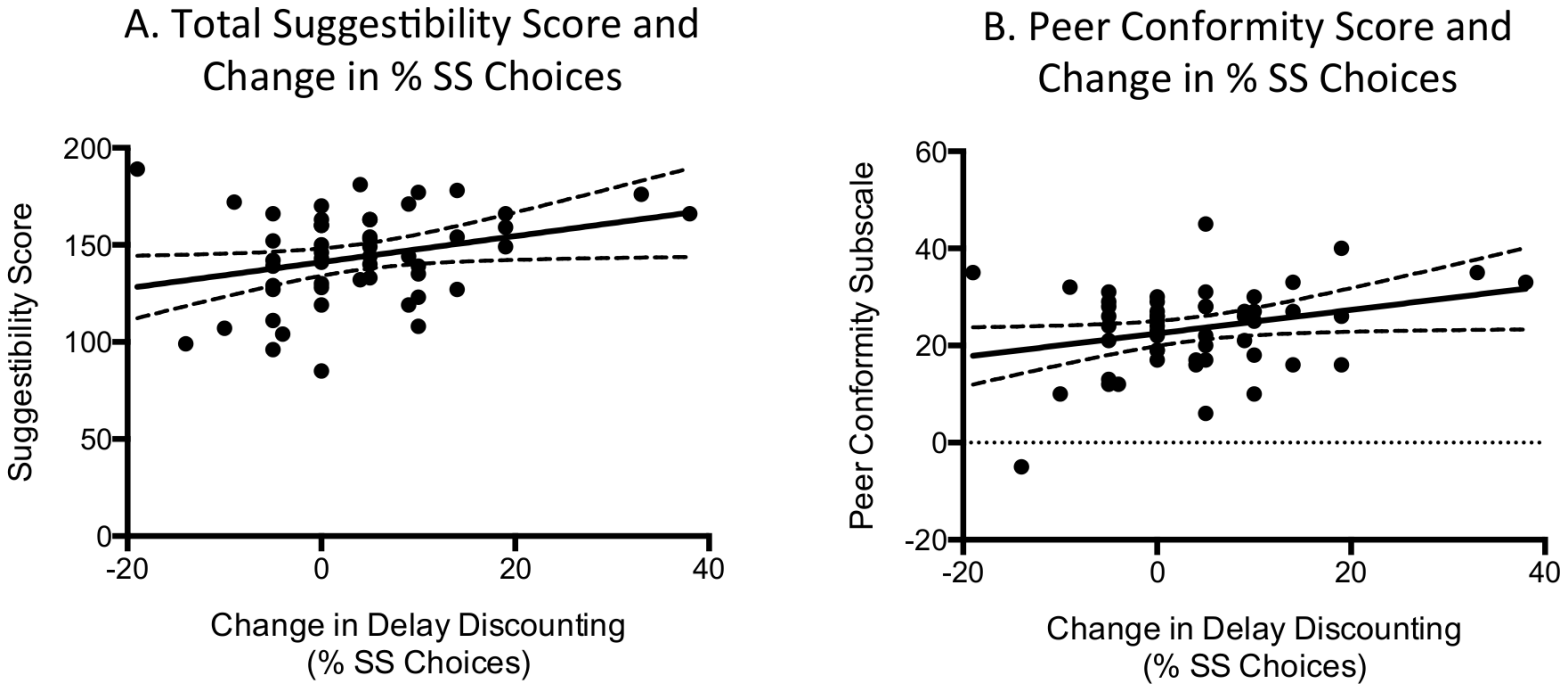
Associations between percentage of SS choices and (A) MISS total suggestibility score and (B) Peer Conformity Subscale score. Dotted lines represent the 95% confidence interval of the associations.

The effect of impulsive vs non-impulsive influence on percent SS choices (calculated as percent SS choices during impulsive minus nonimpulsive influence) correlated with the total score on the MISS (*R* ^2^ = .08, *F*(1, 50) = 4.43, *p* = 0.04; Table 2, Figure 3A). The difference score of percent SS choices was also associated with the peer conformity subscale on the MISS (*R* ^2^ = .08, *F*(1, 50) = 4.30, *p* = 0.04; Figure 3B).

**Table 2:** Association between increases in impulsive choices with impulsive influence and self-reported susceptibility to influence.

Associations with the MISS	*r*	*R* ^2^	*p*
*Change in percent impulsive choices (Impulsive vs. non impulsive influence)*			
MISS Suggestibility Scale Total Score	0.29	0.08	**0.04**
MISS Peer Conformity Subscale Score	0.28	0.08	**0.04**
*Change in K Value (Consistent effect of impulsive influence on responding)*			
MISS Suggestibility Scale Total Score	0.28	0.08	0.07
MISS Peer Conformity Subscale Score	0.28	0.07	0.07

Significant associations are indicated in bold text.

The difference score of log[*k*] values was not significantly associated with the total score on the MISS (*R* ^2^ = .08, *F*(1, 41) = 3.52, *p* = .07). The difference score of log[*k*] values was also not significantly associated the peer conformity subscale on the MISS (*R* ^2^ = .07, *F*(1, 41) = 3.45, *p* = 0.07).

### Association of Delay Discounting Performance with Impulsivity Scores

**Figure 4:**
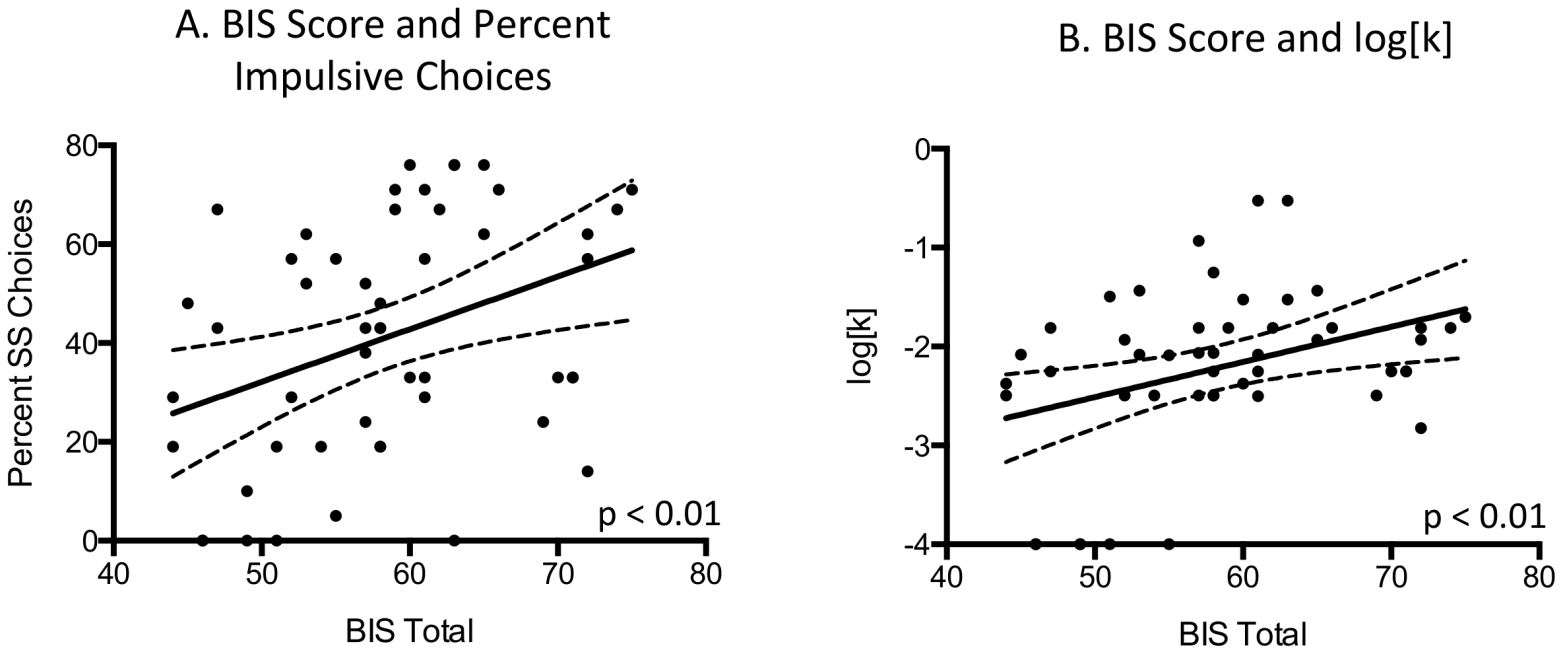
Associations between BIS total score and (A) percentage of impulsive choices, and (B) change in k value, both under the ‘no influence’ condition. There were no slope differences between the ‘no influence’ condition and any influence conditions. Dotted lines represent the 95% confidence interval of the associations.

Under all influence conditions, participants' percentage of impulsive choices and log[*k*] values significantly associated with self-reported impulsivity on the BIS (all r>0.1, all p<0.01)(Figure 4). There were no significant slope differences between associations between the BIS and the four types of influence.

### Analysis of High-and Low Suggestibility and Task Behavior

Those scoring in the top half on the MISS suggestibility scale (i.e. reporting high suggestibility) (n = 25) showed a significant effect of impulsive responding across task conditions (F (2,24) = 6.0, p = 0.01). Post-hoc tests indicated that this effect was driven by impulsive influence; participants had a higher rate of SS choices in decisions with impulsive influence than with non-impulsive, or no influence (p = 0.03). Cohen's *dz* values ranged from 0.53 to 0.54, indicating medium effect sizes in these contrasts (Table 3). Non-impulsive peer influence did not impact decision-making (compared with no influence; *p*>0.1).

**Table 3:** Comparisons in Influence Conditions using In High and Low Suggestibility Groups.

Pairwise Comparisons Influence Type	High Suggestibility Group (n = 25)		Low Suggestibility Group (n = 25)	
	Effect Size (Cohen's *dz*)	Adjusted *p* Value	Effect Size (Cohen's *dz*)	Adjusted *p* Value
***Percentage of Impulsive Choices***				
Impulsive vs. Nonimpulsive	**0.53**	**0.03**	0.13	0.78
Impulsive vs. None	**0.54**	**0.03**	0.07	0.91
Nonimpulsive vs. None	**0.24**	0.47	0.22	0.51

All *p* values were corrected using Tukey's multiple comparison test. Cohen's *dz* values >0.2 and *p* values <0.05 are indicated in bold text.

In contrast, those scoring in the bottom half on the MISS (n = 25) did not show any effect of social influence across conditions (*F* (2,24) = 0.61, *p* = 0.55) (Table 3).

## Discussion

This study demonstrates that delay discounting rates can be modulated by social influence. Using a computerized delay discounting task and a within-subject social influence manipulation, we demonstrated that when young adult participants observed peer responses favoring SS rewards, they were more significantly more likely to select SS rewards themselves. Log[k] values, or discount functions, were not affected by peer influence, indicating that peer influence had a significant effect of random or occasional SS responding, but did not affect more stable patterns of decision-making. Furthermore, performance on this task significantly correlated with self-reported susceptibility on the MISS. This association suggests that task behavior may be related to other domains of social influence, and may be sensitive to individual differences in suggestibility to influence.

While this is the first study to our knowledge to report such a finding in a controlled laboratory setting, it is not surprising that knowledge of impulsive peer choices can affect impulsive decision-making in young adults. Gardner and Steinberg (2005) showed that when mid- and late- adolescents (ages 13–22) participated in a video driving game, either alone or in the presence of two peers, the participants took significantly more risks simply in the presence of peers [34]. Functional MRI studies have shown that adolescents who believe that close friends were watching them play a simulated driving game also exhibited greater ventral striatum activation, which has been shown to correlate with impulsive choices, than those who did not believe they were being observed [45]. Other studies have shown that delay discounting rates are steeper in adolescents who believe they are being observed [32], [33]. The current study extends these results to show that (1) peers do not have to be physically present, or even observing the participants' behavior, to exert influence, and (2) particularly in highly suggestible young adults, the type of influence (i.e. ‘impulsive’ influence or ‘non-impulsive’ influence) matters, in that impulsive influence has an effect, whereas non-impulsive influence may not.

There are various explanations to why social influence would affect participants' choices. A long line of research, called social modeling, argues that people directly adjust their behavior to that of others (e.g. [7]). Social learning theorists hypothesize that individuals form beliefs and attitudes about the behaviors they observe, which impact their own behavior. To our knowledge, this is the first study to directly examine how different types of influence affect delay discounting choices and rates, though previous studies have examined the influence of peers on eating behaviors, drug use, and physical activity. Researchers reported that individuals ate more when their peers ate more, and ate less when their peers ate less [46], [47], [48]. Furthermore, children (ages 9–11) were more likely to choose healthy snacks when unfamiliar peers chose healthy snacks, even in the presence of both healthy and unhealthy snacks [49]. Generally, peoples' perceptions of others' behavior have been found to be strong predictors of behavior, and this is especially true when the “others'” are thought of as a peer group. For example, one study found that college age participants' alcohol intake was more strongly associated with the drinking behavior of their friends, than with the drinking behavior of college students outside their peer group [50]. In another study, norms associated with friends' physical activity was the strongest predictor of an individual's physical activity [51]. Our experiment was optimized to capitalize on this peer effect. Instead of showing photos at random, participants were allowed to choose, out of a set of 32, the young adult ‘peers’ whose answers they would most like to see. Simply selecting these photos may have created an affiliation between the participants and the anonymous photographs.

It is intriguing that among highly susceptible individuals, only impulsive influence had a significant effect on behavior. It is possible that people in general are more swayed by impulsive peers, as these individuals may offer them an opportunity to make a choice outside of the accepted societal norms. It should be noted that participants in this study were generally well-functioning college students from near-optimal environments. It is possible that among individuals from disadvantaged backgrounds, prosocial peer influence may have a greater effect on decision-making (e.g. [4]).

It is also intriguing that the influence on the percentage of impulsive choices was stronger than the influence on *k* values. While percentage of SS choices included all trials in which the participant chose the impulsive option (even inconsistently), a change in the *k* value represented a more consistent switch in the participants' preference. This indicates that the impulsive influence is altering specific responses throughout various impulsivity ranks, but is not altering a more stable measurement of the *k* value. Therefore, it is possible that the participants' core preferences were not significantly changing, but the social influence manipulation was sufficient to cause them to change their responses during at least some trials, in an inconsistent manner.

The change in percentage of SS choices was associated with self-reported susceptibility indices on the MISS. There are limitations to self-report measures, as research participants under-report behaviors deemed inappropriate by researchers or other observers, and over-report behaviors viewed as appropriate (see [9] for review). Therefore, laboratory social manipulations present an opportunity to more accurately assess susceptibility to influence, and may offer insight into subtle changes in susceptibility that may not even be evident to the participant. Further, this association also indicates that some individuals are more easily influenced than others. About a third of participants demonstrated strong social influence effects. This is consistent with classical studies of social influence (e.g. [52], [53], [54]), in which subjects were asked to make a perceptual judgment. When participants were isolated, the rate of correct responses was close to 100%. When participants were part of a group consisting of confederates who would give the wrong answer, 76% of the participants went along with the group and selected the wrong answer at least once [52] Asch identified two subtypes of people, whom he referred to as “independent” (26% of participants who never agreed with the majority) and “yielding” (28% who agreed on more than half of all trials), and concluded that some individuals were more likely to be influenced by groups than others.

There are several limitations to this preliminary study that should be noted. First, all participants in this study were young adults (ages 18–25); future research should examine both younger adolescents or older adults in order to investigate whether social influence on discounting rates changes across the lifespan. Second, future work with larger sample sizes can delve deeper into factors that may determine an individual's social influence susceptibility, such as their age, ethnicity, gender, and other personality characteristics. It would also be interesting to determine whether different peer groups (i.e. those in the same age group or the same gender) would exert a larger effect on behavior. It is also not known how viewing faces during the task would itself affect *k* values, though the estimated *ks* from the ‘no influence’ condition are within range of the reported *k* values in the literature for this age group [35], indicating that adding faces alone did not change overall performance. We used a hypothetical task, which may have limited relevance to real-world consequences; though there is literature demonstrating that hypothetical monetary rewards produce results comparable to those obtained with real monetary rewards (e.g. [42], [43]), future studies could compare this task to one with actual rewards. Finally, this study used static images to elicit influence, and it is possible that bringing in actual peers may have affected the data in this study.

In summary, this study utilized a novel social influence delay discounting paradigm to demonstrate that social influence affects impulsive decision-making in young adults. In highly suggestible participants, only impulsive social influence increased impulsive decision-making, while non-impulsive influence did not reduce impulsive decision-making. These effects are highly associated with self-reported susceptibility to influence, indicating that this novel manipulation to a well-validated task may be useful in objectively assessing susceptibility to peer influence.

## Supporting Information

Table S1 **Individual Raw and Log-Transformed** ***k*** **values and Consistency Scores for Delay Discounting.**
(DOCX)
