# Supplementary material for: Impulsive Social Influence Increases Impulsive Choices on a Temporal Discounting Task in Young Adults
Source: PLoS One. 2014 Jul 2;9(7):e101570. doi: 10.1371/journal.pone.0101570 (PMC4079280; doi:10.1371/journal.pone.0101570)
Supplement: Table S1 — Individual Raw and Log-Transformed k values and Consistency Scores for Delay Discounting. (DOCX) [file pone.0101570.s001.docx]

Supplementary Table 1. Individual Raw and Log-Transformed *k* values and Consistency Scores for Delay Discounting

|  | No Influence | | | Impulsive Influence | | | Non-impulsive Influence | | |
| --- | --- | --- | --- | --- | --- | --- | --- | --- | --- |
| Participant ID | *k* | log[*k*] | Consistency | k | log[*k*] | Consist-ency | *k* | log[*k*] | Consist-ency |
| 1 | 0.0015 | -2.8249 | 0.95 | 0.0015 | -2.8249 | 0.95 | 0.0032 | -2.4949 | 1.00 |
| 2 | a |  | 1.00 | a |  | 0.95 | a |  | 1.00 |
| 3 | 0.0199 | -1.7003 | 1.00 | 0.0199 | -1.7003 | 0.95 | 0.0199 | -1.7003 | 0.90 |
| 4 | 0.0081 | -2.0895 | 0.90 | 0.0083 | -2.0821 | 0.90 | 0.0056 | -2.2519 | 0.95 |
| 5 | 0.0042 | -2.3764 | 0.90 | 0.0056 | -2.2519 | 0.95 | 0.0032 | -2.4949 | 0.95 |
| 6 | 0.0081 | -2.0895 | 0.81 | 0.0056 | -2.2519 | 0.95 | 0.0056 | -2.2519 | 0.95 |
| 7 | 0.0117 | -1.9307 | 0.86 | 0.0367 | -1.4354 | 0.95 | 0.0083 | -2.0821 | 0.86 |
| 8 | 0.0086 | -2.0658 | 0.90 | 0.0042 | -2.3764 | 0.95 | 0.0086 | -2.0658 | 0.95 |
| 9 | 0.0015 | -2.8249 | 0.95 | 0.0015 | -2.8249 | 0.90 | 0.0032 | -2.4949 | 1.00 |
| 10 | 0.0042 | -2.3764 | 0.95 | 0.0086 | -2.0658 | 1.00 | 0.0068 | -2.1703 | 0.95 |
| 11 | 0.0086 | -2.0658 | 0.81 | 0.1310 | -0.8827 | **0.67** ^b^ | 0.0015 | -2.8249 | 0.76 |
| 12 | 0.0707 | -1.1506 | 0.90 | 0.0367 | -1.4354 | 1.00 | 0.0199 | -1.7003 | 1.00 |
| 13 | 0.0300 | -1.5235 | 0.90 | 0.0081 | -2.0895 | 0.90 | 0.0083 | -2.0821 | 0.90 |
| 14 | 0.0042 | -2.3764 | 1.00 | 0.0083 | -2.0821 | 0.95 | 0.0056 | -2.2519 | 0.95 |
| 15 | 0.0117 | -1.9307 | 0.81 | 0.0056 | -2.2519 | 0.90 | 0.0032 | -2.4949 | **0.71** ^b^ |
| 16 | 0.0300 | -1.5235 | 0.95 | 0.0199 | -1.7003 | 0.95 | 0.0199 | -1.7003 | 1.00 |
| 17 | 0.0199 | -1.7003 | 0.86 | 0.0154 | -1.8113 | 0.86 | 0.0199 | -1.7003 | 0.90 |
| 18 | 0.0117 | -1.9307 | 0.81 | 0.0367 | -1.4354 | 0.95 | 0.0707 | -1.1506 | 0.95 |
| 19 | 0.0083 | -2.0821 | 0.95 | 0.0083 | -2.0821 | 0.90 | 0.0042 | -2.3764 | 0.90 |
| 20 | 0.0015 | -2.8249 | 0.81 | 0.0015 | -2.8249 | 0.90 | 0.0093 | -2.0318 | 0.95 |
| 21 | 0.0086 | -2.0658 | 0.90 | 0.0086 | -2.0658 | 0.95 | 0.0086 | -2.0658 | 0.90 |
| 22 | 0.0015 | -2.8249 | 0.95 | 0.0015 | -2.8249 | 0.95 | 0.0032 | -2.4949 | 1.00 |
| 23 | 0.0086 | -2.0658 | 0.86 | 0.0086 | -2.0658 | 0.86 | 0.0086 | -2.0658 | 0.95 |
| 24 | 0.0001 | -4.0000 | 1.00 | 0.0007 | -3.1549 | 1.00 | 0.0007 | -3.1549 | 1.00 |
| 25 | 0.0081 | -2.0895 | 0.86 | 0.0081 | -2.0895 | 0.90 | 0.0083 | -2.0821 | 1.00 |
| 26 | 0.0117 | -1.9307 | 0.90 | 0.0081 | -2.0895 | 0.95 | 0.0154 | -1.8113 | 0.95 |
| 27 | a |  | 1.00 | a |  | 1.00 | a |  | 1.00 |
| 28 | 0.0300 | -1.5235 | 0.90 | 0.0117 | -1.9307 | 0.86 | 0.0707 | -1.1506 | 0.90 |
| 29 | 0.0300 | -1.5235 | 0.95 | 0.0199 | -1.7003 | 1.00 | 0.0199 | -1.7003 | 0.95 |
| 30 | 0.0117 | -1.9307 | 0.95 | 0.0154 | -1.8113 | 0.90 | 0.0154 | -1.8113 | 0.90 |
| 31 | 0.0068 | -2.1703 | 0.90 | 0.0015 | -2.8249 | 0.90 | 0.0032 | -2.4949 | 0.95 |
| 32 | 0.0199 | -1.7003 | 0.86 | 0.0367 | -1.4354 | **0.76** | 0.0199 | -1.7003 | 0.95 |
| 33 | 0.0015 | -2.8249 | 0.95 | 0.0015 | -2.8249 | 0.95 | 0.0015 | -2.8249 | 0.95 |
| 34 | 0.0007 | -3.1549 | 1.00 | 0.0015 | -2.8249 | 1.00 | 0.0007 | -3.1549 | 1.00 |
| 35 | 0.0015 | -2.8249 | 0.90 | 0.0015 | -2.8249 | 0.90 | 0.0032 | -2.4949 | 0.95 |
| 36 | 0.0117 | -1.9307 | 0.90 | 0.0117 | -1.9307 | 0.90 | 0.0117 | -1.9307 | 0.90 |
| 37 | a |  | 1.00 | a |  | 1.00 | a |  | 1.00 |
| 38 | 0.0117 | -1.9307 | 0.95 | 0.0081 | -2.0895 | 0.90 | 0.0154 | -1.8113 | 0.90 |
| 39 | 0.0086 | -2.0658 | 0.95 | 0.0081 | -2.0895 | 0.90 | 0.0086 | -2.0658 | 0.90 |
| 40 | 0.0086 | -2.0658 | 1.00 | 0.0086 | -2.0658 | 0.95 | 0.0056 | -2.2519 | 1.00 |
| 41 | 0.0086 | -2.0658 | 0.90 | 0.0086 | -2.0658 | 0.95 | 0.0056 | -2.2519 | 1.00 |
| 42 | 0.0086 | -2.0658 | 0.90 | 0.0086 | -2.0658 | 0.95 | 0.0086 | -2.0658 | 0.86 |
| 43 | a |  | 1.00 | a |  | 1.00 | a |  | 1.00 |
| 44 | 0.0117 | -1.9307 | 1.00 | 0.0199 | -1.7003 | 0.86 | 0.0117 | -1.9307 | 0.90 |
| 45 | 0.0032 | -2.4949 | 0.48^b^ | 0.0199 | -1.7003 | 0.81 | 0.0056 | -2.2519 | 0.76 |
| 46 | 0.0007 | -3.1549 | 0.90 | 0.0042 | -2.3764 | 0.90 | 0.0007 | -3.1549 | 1.00 |
| 47 | 0.0367 | -1.4354 | 0.86 | 0.0367 | -1.4354 | 0.81 | 0.0086 | -2.0658 | 0.76 |
| 48 | 0.0015 | -2.8249 | 0.90 | 0.0015 | -2.8249 | 0.90 | 0.0056 | -2.2519 | 0.95 |
| 49 | 0.0300 | -1.5235 | 0.95 | 0.0154 | -1.8113 | 0.90 | 0.0199 | -1.7003 | 0.95 |
| 50 | 0.0300 | -1.5235 | 0.90 | 0.0367 | -1.4354 | 0.86 | 0.0032 | -2.4949 | **0.71** ^b^ |
| 51 | 0.0015 | -2.8249 | 0.95 | 0.0042 | -2.3764 | 0.95 | 0.0032 | -2.4949 | 1.00 |
| Mean | 0.0123 | -2.1671 |  | 0.0145 | -2.1031 |  | 0.0111 | -2.1713 |  |
| SD | 0.0131 | 0.5570 |  | 0.0206 | 0.4975 |  | 0.0141 | 0.4433 |  |
| Skewness | 2.3205 | -0.9146 |  | 4.2095 | -0.1115 |  | 3.3976 | -0.1808 |  |
| Kurtosis | 7.5327 | 1.2337 |  | 22.4699 | -0.2749 |  | 12.8047 | 0.5462 |  |

^a^The value of k could not be calculated because the participant chose the delayed choice every time.

^b^ A consistency score < .75 indicates that the parameter estimates for the k value may not be a good fit because of the participant’s lack of consistent responding.
